# Digital advance care planning education in nursing home placements: a qualitative descriptive study of nursing students’ experiences

**DOI:** 10.1186/s12904-026-02248-0

**Published:** 2026-07-22

**Authors:** Stephanie Craig, Julie Doherty, Kevin Brazil, Gary Mitchell

**Affiliations:** School of Nursing and Midwifery, Queen’s University Belfast, Belfast, Northern Ireland

**Keywords:** Advance care planning, Palliative care, Nursing students, Undergraduate nursing education, Nursing homes, Digital education, E-learning, Self-efficacy, Qualitative research, Clinical placement

## Abstract

**Background:**

Advance care planning (ACP) is a core component of high-quality palliative and end-of-life care, particularly in nursing homes where residents commonly experience frailty, multimorbidity and cognitive impairment. Although digital ACP education has shown promise for qualified staff, limited qualitative evidence has examined how undergraduate nursing students experience such learning during clinical placement. This study explored first-year nursing students’ experiences of using an online e-resource during nursing home placements exploring its acceptability, perceived influence on knowledge and confidence, and application to practice.

**Methods:**

A qualitative descriptive study was undertaken within a UK School of Nursing and Midwifery. All eligible first-year undergraduate nursing students undertaking nursing home placements between January and March 2024 received access to a care-home-specific ACP e-resource. Sixteen students participated in semi-structured online interviews. Data were analysed using reflexive thematic analysis, informed by Social Cognitive Theory, particularly self-efficacy.

**Results:**

Three interrelated themes were identified: Building Confidence, Putting Learning into Practice, and Spreading the Learning. Students described the resource as accessible, relevant to the nursing home context, and appropriately pitched to their stage of learning. It appeared to support greater perceived readiness to recognise and engage with ACP-related issues in practice, including discussion of deterioration, future care and end-of-life planning. Students also valued acknowledgement of the emotional impact of care, particularly the inclusion of self-care content. Although participants identified limitations of asynchronous learning and expressed a preference for greater rehearsal and interactivity, many also described sharing the resource with staff, peers and others in practice settings.

**Conclusions:**

A digital, care-home-specific e-resource may support undergraduate nursing students’ preparedness for palliative and end-of-life communication during nursing home placements. Embedding context-specific digital ACP education within preregistration curricula may strengthen early preparedness, particularly when supplemented by opportunities for reflection, rehearsal and discussion.

## Background

Advance care planning (ACP) is a core component of high-quality palliative and end-of-life care [1]. It enables individuals to identify their values, consider goals for future treatment, and communicate care preferences should they lose decision-making capacity [2]. ACP is an ongoing, relational process involving patients, families and healthcare professionals, ideally initiated early and revisited over time [3, 4]. By aligning clinical decisions with an individual’s wishes, ACP supports person-centred care, may reduce unwanted interventions and enhance quality of care [5].

ACP is especially important in nursing homes, where many residents live with advanced frailty, multimorbidity and dementia [6, 7]. Progressive cognitive decline may limit residents’ ability to express preferences regarding life-sustaining treatment or comfort-focused care, increasing reliance on families and healthcare professionals to make proxy decisions [2, 8]. Family carers may feel uncertain or underprepared for end-of-life decision-making, while nursing home staff can lack confidence in initiating conversations about deterioration, dying and future care [9, 10]. Strengthening ACP capability in nursing homes therefore remains an international palliative care priority [1].

Educational interventions to improve ACP knowledge and communication skills have shown promise [11]. Online ACP training for nursing home staff has been associated with improved knowledge, confidence and willingness to engage in end-of-life conversations [10], while structured education for nursing students and other health professionals has also reported gains in knowledge, confidence and perceptions of end-of-life care [12, 13]. However, most evidence has focused on qualified staff, classroom-based teaching or simulation, with less attention to students’ experiences during clinical placement.

Nursing home placements expose undergraduate nursing students to residents with advanced illness, frailty and dementia early in training [14]. Although these settings provide opportunities for experiential learning, they can also present emotional and ethical challenges, particularly around deterioration, death and sensitive communication with residents and families [15]. Competence in ACP requires knowledge of policy and legal frameworks, communication skills, ethical reasoning and confidence to engage sensitively in practice [16]. Integrating ACP education into preregistration curricula is therefore important in preparing future nurses for palliative care in long-term care settings [17].

Digital education is increasingly used across health professions education [18–23]. Technology-supported learning offers flexibility, accessibility and self-paced engagement, which may benefit students balancing academic study with clinical placements [24–26]. Systematic reviews suggest that e-learning can enhance knowledge and self-reported confidence, particularly when supported by clear learning objectives and interactive components [27, 28]. Digital approaches, including serious games, podcasts and simulation technologies, may also support knowledge translation and connect theoretical learning with practice [18, 21, 29–31].

Despite growing interest in digital interventions in palliative care [32] and dementia education [19, 33], limited qualitative evidence has examined how undergraduate nursing students experience online ACP learning during nursing home placements. Little is known about how such resources shape perceived confidence, readiness for sensitive communication and application of learning in practice. These outcomes align with Bandura’s Social Cognitive Theory, particularly self-efficacy, which proposes that people are more likely to undertake challenging tasks when they believe they can perform them effectively [34–36]. Digital education may support self-efficacy through structured knowledge, communication models and opportunities for cognitive rehearsal before or during placement exposure [37].

This study aimed to explore first-year undergraduate nursing students’ experiences of using an online ACP e-resource during nursing home placements, with particular attention to perceived knowledge development, confidence and application of learning in practice.

## Methods

### Study design

A qualitative descriptive study was undertaken. Qualitative description is well suited to applied healthcare research where the aim is to generate practice-relevant findings grounded in participants’ accounts rather than highly abstract theoretical interpretation [38–40].

### Theoretical framework

The study was informed by Social Cognitive Theory, particularly self-efficacy, as a sensitising framework. This informed development of the semi-structured interview guide by shaping questions and prompts relating to students’ perceived confidence, preparedness, and readiness to engage with ACP in practice. During analysis, reflexive thematic analysis remained primarily inductive; however, interpretation was theoretically sensitised to participants’ accounts of perceived capability, reduced apprehension, and readiness to apply learning in placement.

The study was informed by an interpretivist orientation, recognising that participants’ accounts were shaped by context, experience and interaction [41, 42]. Social Cognitive Theory, particularly self-efficacy, served as a sensitising framework for interview design and for interpreting patterns relating to confidence, preparedness, and perceived capability in practice [35]. The study is reported in accordance with the Consolidated Criteria for Reporting Qualitative Research (COREQ) [43].

### Setting and participants

The study was conducted within a single School of Nursing and Midwifery at a UK university. Eligible participants were first-year undergraduate nursing students scheduled to undertake a nursing home placement between January and March 2024. Students had received introductory teaching on professional communication, person-centred care and caring for older adults, but had not received dedicated teaching on ACP. Previous exposure to palliative and end-of-life care varied according to personal, employment and placement experiences. For many students, the nursing home placement therefore represented an early opportunity to encounter deterioration, future care planning and end-of-life care in practice.

A pragmatic convenience sample was used because nursing home placements at the host institution are most commonly allocated during first year and occur less frequently in later years, resulting in a cohort with broadly comparable clinical exposure. Students were eligible if they were enrolled in the undergraduate nursing programme, scheduled to complete a nursing home placement during the study period, and had received access to the e-resource before placement.

Before placement, all eligible students (*n* = 49) were provided with access to the e-resource and invited to engage with it during protected time in the first week, as agreed with placement partners. The university placement office acted as gatekeeper and emailed all eligible students to ask whether they were willing to be contacted about an interview. Participation was voluntary.

Sample adequacy was considered using the concept of information power [44]. The study had a narrow and specific aim, and participants were relatively homogeneous in relation to stage of training and placement context. Social Cognitive Theory, particularly self-efficacy, sensitised the study, while in-depth semi-structured interviews lasting 22–48 min generated rich experiential accounts. Collectively, these factors indicated that the sample provided sufficient information power to address the study aim.

### Educational E-resource

Participants received access to the *Necessary Discussions ACP Care Homes* e-resource, an online educational programme developed for care home staff and focused on ACP principles, communication and documentation in care home practice [9, 10]. It comprised six brief units covering: an introduction to ACP; ACP processes and participation; completing an advance care plan; recording and sharing plans; communication strategies for necessary discussions; and self-care when engaging in sensitive conversations. Content was delivered through short articles, videos and reflective activities and was designed for self-paced learning.

All eligible students were given access before placement. The resource was intended to take approximately 1.5–2 h to complete, and placement partners were asked to provide up to two hours of protected time during the first week. Completion was encouraged but not mandatory, and students could engage with all or part of the resource.

Objective engagement data, including time spent, unit completion and overall completion rates, were not collected. The provision of protected time was also not formally monitored across placements. It was therefore not possible to determine the extent of individual engagement or the consistency with which protected time was implemented. Findings relating to use of the resource are consequently based on participants’ interview accounts.

### Data collection

Data were collected between January and March 2024 through individual semi-structured interviews conducted by GM. The interview guide was developed collaboratively and informed by the study aim, literature on digital education and ACP, and sensitising concepts from Social Cognitive Theory [35]. Questions explored students’ experiences of the e-resource, its perceived influence on knowledge and confidence, application during placement, and sharing of learning with nursing home staff (Table 1).

**Table 1:** Study interview guide

Topic area	Main question	Possible prompts
Experience of the resource	Can you tell me about your experience of using the e-resource during placement?	How much did you use it? What were your first impressions? Was it easy to use? Which parts stood out most?
Learning and confidence	Did the resource influence what you knew or how confident you felt about ACP?	Did it change your understanding of ACP? Did it make conversations seem easier or less intimidating? Did it help you feel more prepared?
Use in practice	Did you use anything from the resource during your nursing home placement?	Did it affect how you spoke with residents or staff? Did it help you recognise ACP in practice? Were there times you wanted to use it but could not?
Sharing and reflections	Did you discuss or share the resource, or what you learned from it, with anyone else?	With staff or other students? How was it received? What was most useful? What could be improved?

Interviews were conducted via Microsoft Teams while participants were in a private room within the nursing home during placement hours. They lasted 22–48 min, were audio-recorded, transcribed verbatim [45, 46], and checked against the recordings for accuracy before analysis.

### Data analysis

Data were analysed using reflexive thematic analysis as described by Braun and Clarke [47]. Analysis was primarily inductive, while remaining theoretically sensitised to issues of confidence, preparedness and self-efficacy. The analytic process involved repeated familiarisation with the transcripts, generation of initial codes, development of candidate themes, review and refinement of themes, and definition and naming of final themes.

GM and SC were actively involved in coding, interpretation and theme development, with themes refined through ongoing reflexive discussion within the research team. During coding, GM and SC compared interpretations and discussed areas where extracts could be read in different ways. For example, some data initially coded as confidence-building were reconsidered as relating more specifically to relevance, accessibility, protected time and emotional support. These were retained within Theme 1 but interpreted as conditions that helped make ACP feel more understandable and manageable for first-year students, rather than as direct evidence of confidence itself. The analysis focused primarily on patterns within participants’ accounts, while also considering how students described confidence, readiness and the perceived application of learning in practice.

### Reflexivity and trustworthiness

GM, KB and JD were involved in the original Necessary Discussions project in nursing home settings. The present study extended that work into nursing education and was led by GM and SC, who undertook the data collection and analysis. SC had not been involved in the earlier project and therefore contributed a distinct perspective during analysis. GM was known to participants in an academic capacity and had led implementation of the resource within nurse education. Reflexive discussions were undertaken throughout data collection and analysis to consider how researchers’ prior assumptions, professional experience and familiarity with the e-resource may have shaped interpretation [48]. Consistent with a reflexive thematic approach, the research team’s familiarity with the e-resource was viewed not only as a potential influence on interpretation but also as a source of contextual insight that informed analytic understanding and meaning-making. Rigour was supported through verbatim transcription, iterative theme development, maintenance of an audit trail, and clear description of the study context and participants to support transferability [49].

To minimise the potential influence of pre-existing relationships and assumptions, participants were reminded at the outset of each interview that there were no right or wrong answers and that both positive and negative experiences of the e-resource were equally valuable to the study. Open-ended questioning and probing techniques were used to encourage participants to elaborate on their experiences and to discuss aspects of the resource that they found helpful, challenging or less useful. During analysis, reflexive discussions were used to critically examine interpretations and consider how the team’s proximity to the e-resource may have shaped analytic decisions. SC’s position outside the original project encouraged consideration of alternative interpretations, including whether students’ descriptions of sharing the e-resource reflected its perceived value or routine placement-based conversations about learning. Participants did offer critical accounts, including concerns about the passive nature of asynchronous learning, variable motivation to engage, limited opportunities for rehearsal, and continued anxiety about applying ACP communication in practice.

## Ethical considerations

Ethical approval was obtained from the Faculty of Medicine, Health and Life Sciences Research Ethics Committee at Queen’s University Belfast (Reference: MHLS22_111). All procedures were conducted in accordance with the Declaration of Helsinki [50]. Informed written consent was obtained from all participants prior to data collection. Participation was voluntary, and students were assured that involvement or non-participation would not influence academic progression.

## Results

Thirty-one students indicated willingness to be contacted, and sixteen subsequently completed an interview (This represented 33% of the 49 eligible students). Of the remaining 15 students, 12 did not respond to follow-up emails to arrange an interview, two were unable to attend because of placement demands and did not rearrange, and one left placement early because of illness. Participant characteristics are presented in Table 2.

**Table 2:** Participant characteristics

Characteristic	Category	*n* (%)
Total participants		16 (100)
Gender	Female	12 (75.0)
	Male	4 (25.0)
Field of nursing	Adult nursing	10 (62.5)
	Learning disability nursing	4 (25.0)
	Mental health nursing	2 (12.5)
Age group	18–25 years	13 (81.3)
	30–35 years	1 (6.3)
	40–45 years	1 (6.3)
	46–50 years	1 (6.3)

Analysis generated three interrelated themes (1) Building Confidence, (2) Putting Learning into Practice and (3) Spreading the Learning. These themes described how students experienced, used, and shared learning from the online e-resource during nursing home placement.

The first theme, Building Confidence, captures how students engaged with the resource as a manageable, relevant and emotionally attuned form of learning. Participants valued its fit with the nursing home context, the protected time given for completion, and the way it made ACP feel more understandable at an early stage of training. At the same time, they identified limitations of asynchronous learning and expressed a desire for more interactive elements.

The second theme, Putting Learning into Practice, describes how students moved from learning about ACP to recognising and using it in placement. Students reported greater awareness of ACP in everyday care, increased willingness to discuss deterioration and future care, and examples of participation in care planning discussions.

The third theme, Spreading the Learning, reflects how the resource was often shared beyond the student learner. In several placements, students described introducing the resource to staff, managers, peers and occasionally family members, extending its reach within and beyond the nursing home setting.

**Fig. 1:**
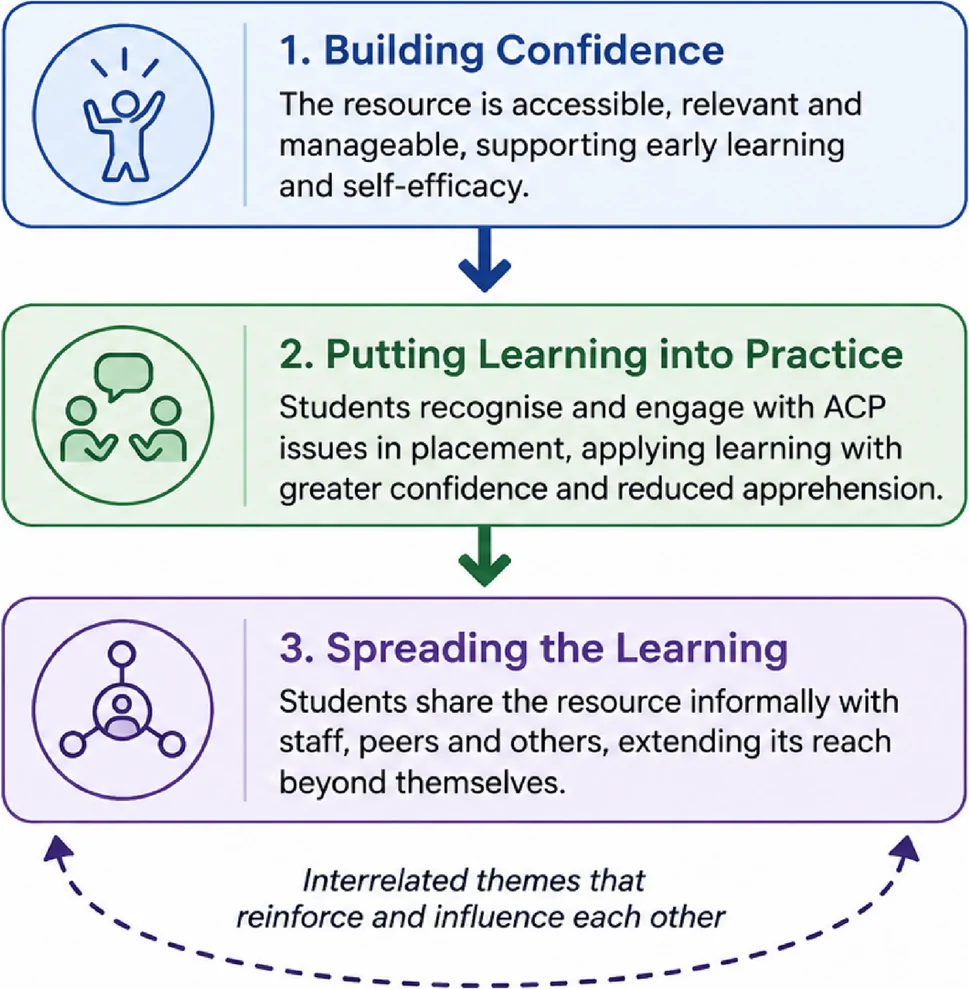
Relationship and progression across the three themes

Figure 1 illustrates the relationships among these themes, which present a progression from building confidence to applying learning in practice to sharing learning with others, while recognising that the themes are interrelated rather than strictly linear.

### Theme 1: building confidence

Students described confidence-building as being facilitated by several interconnected aspects of the e-resource, including its relevance to nursing home practice, its accessible and manageable structure, opportunities to engage with the resource during placement, and content that supported preparedness for ACP-related conversations.

Students consistently described the resource as learning that felt closely connected to the setting in which they were placed. Rather than experiencing ACP as a distant or abstract topic, participants said the resource felt directly relevant to the nursing home environment and to the residents they were caring for. Participants’ recognition of the relevance and importance of ACP appeared to create conditions that supported confidence-building. By seeing direct links between the resource content and their placement experiences, students described the material as more meaningful, approachable and immediately applicable to practice.

*It felt like it was actually about the place I was in*,* not just general information that you maybe read and think*,* right*,* that’ll be useful some day [P2].*

Another explained,

*Because I was seeing residents every day and hearing staff talk about future care*,* it made sense straight away why this mattered [P7].*

The way the resource was structured also supported this sense of accessibility. Students described the content as broken into manageable sections, which helped make ACP feel less overwhelming. Several participants spoke about the value of short units, simple explanations and a clear flow through the topic. For learners in the early stages of training, this seemed to reduce the sense that ACP was too complex or too advanced for them.

*It didn’t feel like another huge bit of work that you’d have to sit and force yourself through. It was broken up well and you could actually take it in [P11].*

Participants also described the clear explanations as making ACP accessible and appropriate for first-year students. Students therefore described the resource not only as understandable, but as pitched at a level that allowed them to engage with a challenging topic without feeling excluded by it.

Several participants linked their engagement with the resource to having protected time during placement to complete it. This protected time was described as more than a practical convenience. Students said it signalled that the learning mattered and that ACP was an important part of placement rather than something extra to be done in their own time. For some, having time set aside made them more likely to use the resource and take it seriously.

*If we had just been sent the link and told to look at it whenever*,* I’m honestly not sure everybody would have done it. But because there was actual time put aside for it*,* it felt like this was something we were meant to pay attention to [P9].*

In this way, participants linked their engagement not only to the content itself but also to the conditions in which the learning was offered.

The asynchronous format was generally viewed positively. Students appreciated being able to work through the material at their own pace and return to sections when needed. This was particularly important during placement, where students were balancing learning, new routines and the demands of practice. Several participants described the resource as something they could “fit in” without it feeling burdensome. The flexibility of online access appeared to support this. At the same time, students were clear that asynchronous learning did not suit everyone equally well. Some reflected that self-directed learning depended on personal motivation and that it could be easy to postpone engagement when placement felt busy or tiring.

*You do know it matters*,* but there’s always so much else going on as well*,* so if you’re tired or there’s loads happening on placement it could be easy enough to leave it until later [P14].*

Another commented,

*It probably won’t suit everyone because some people need more direction*,* or more of that classroom way of learning where somebody is taking you through it [P4].*

These accounts showed that flexibility was valued, but was not without limits.

Students were also positive about the design and presentation of the website. They described it as straightforward to navigate and appreciated the use of different formats, including written content, videos and reflective elements. This variety was said to help maintain attention and support understanding. Some students also commented that the resource did not feel overly simplified despite being accessible. Although it had originally been designed for care home staff, participants generally did not find it too advanced. Instead, some described this as reassuring. Being able to engage with content intended for practice staff appeared to strengthen their sense that ACP was something they could begin to understand as student nurses.

*I liked that it wasn’t dumbed down*,* but it still made sense. It made me feel like maybe I do know a bit more than I think I do [P1].*

Another noted,

*It was for nurses already working there*,* but I never felt it was beyond me or that I shouldn’t be looking at it as a student [P12].*

A particularly memorable part of the resource for several students was the inclusion of self-care content. Participants described this as unexpectedly important. In nursing home placements, students were often encountering decline, dying and emotional conversations for the first time. They valued that the resource acknowledged the emotional effect this work can have, rather than focusing only on communication or documentation.

*That part stood out because you don’t always get told how to look after yourself when people are dying or when families are upset. It was good that it was actually mentioned [P8].*

Another reflected,

*It was good that it recognised this can affect you too*,* especially when you’re new to placement and maybe you haven’t seen much of that before [P15].*

These accounts showed that students experienced the resource as responsive not only to clinical learning needs, but also to the emotional realities of care home practice.

Alongside these positive views, participants also identified ways in which the learning experience could be improved. Several students felt that, although the content was useful, the format could at times feel passive. They appreciated the videos and reflective features, but wanted more opportunities to actively test their understanding or practise responses. This was expressed not as criticism of the resource overall, but as a suggestion for how it might become even more engaging.

*The videos were good*,* but I think maybe something where you had to do a task or choose what you would say would make it stick more [P6].*

Overall, this theme shows that students experienced the resource as relevant, manageable and confidence-building, while also recognising the limits of self-directed digital learning and the value of more active forms of engagement.

### Theme 2: putting learning into practice

Students’ accounts indicated that the resource was not experienced only as something to read or complete, but as something that shaped how they understood and responded to practice. A common feature across interviews was that students described becoming more aware of ACP within the day-to-day life of the nursing home. Some explained that before using the resource they might have noticed conversations or decisions taking place without fully understanding what they were. After engaging with the material, they described being better able to recognise ACP as part of ongoing care.

*Before it*,* I think I would have noticed conversations were happening but not really understood what they were about or why they mattered. Afterwards I could actually see ACP in practice a lot more clearly [P3].*

This suggests that the resource gave students a framework for recognising ACP in the placement setting, rather than leaving it as an unfamiliar or hidden part of care.

Students also described the resource as helping them feel more prepared to speak or contribute when ACP-related issues arose. They did not describe becoming experts or leading discussions independently. Instead, they spoke about feeling more able to participate in appropriate ways, whether through asking questions, noticing issues, or contributing observations. In several interviews, participants gave examples of being present during care planning discussions, observing conversations about anticipatory medications, or being involved in aspects of ACP alongside staff. In one case, a student described suggesting to staff that a family meeting might be useful for a newly admitted resident. Another spoke about having a clearer sense of why certain conversations were happening and feeling more confident in joining them.

*I wasn’t running the conversation or anything like that*,* but I felt I had enough understanding to say this might be something to think about [P10].*

Another said,

*It gave me confidence to speak up a bit more because I understood why it mattered and what the conversation was trying to do [P13].*

Participants repeatedly described the resource as reducing some of the uncertainty surrounding ACP. This did not mean the topic stopped feeling sensitive. Rather, students spoke about it becoming more structured and less intimidating. For some, the resource appeared to make difficult conversations feel more approachable by giving them language and examples that they could draw on.

*It kind of took away some of the fear around saying the wrong thing. I still knew it was a sensitive topic*,* but it felt a bit less like something you should avoid [P16].*

Another commented,

*I wouldn’t say I suddenly felt totally confident*,* but I was definitely less afraid of the topic than before*,* and that was a big change for me [P5].*

These accounts suggest that students experienced a shift from avoidance towards greater readiness to engage, even where uncertainty remained.

This change was described not only in relation to formal care planning processes but also in relation to everyday communication. Students reported being more open to discussing deterioration, dying and future care in a way that felt calmer and more considered. Some suggested that the resource had changed how they thought about these conversations, making them seem less dramatic or taboo and more a part of compassionate nursing care.

*It made me realise these conversations don’t always have to be really dramatic or frightening. Sometimes they can actually be quite calming if they help people understand what’s going on [P6].*

Students’ comments therefore suggested that the resource influenced both what they noticed in practice and how they approached sensitive communication.

For some, the resource also acted as a starting point for further learning. A small number of students described following links, reading additional materials or exploring related sources after completing the initial units. This indicated that the resource was, for at least some participants, a prompt for continued interest rather than a self-contained exercise.

*It made me want to look up more because once you understood the basics*,* you realised there was a lot more to it than I first thought [P7].*

This points to the possibility that the resource helped students see ACP as a meaningful area of practice worth learning more about.

At the same time, participants remained realistic about the limits of what the resource could achieve. They were clear that real conversations with residents and families were still difficult and that online learning could not remove all anxiety. Several students emphasised that knowing the principles of ACP was different from handling emotionally complex situations in practice.

*It definitely helped*,* but I still think I’d be anxious in certain situations*,* especially if family emotions were high or if somebody was very upset [P9].*

Another reflected,

*You can learn the theory online*,* but doing it with real people is different. You still have to work out how to say things in the moment [P14].*

These comments show that students positioned the resource as useful preparation rather than a complete answer to the challenges of ACP communication.

This was reflected in a recurring suggestion that the resource could be strengthened by an added practical component. Across interviews, students described wanting some form of simulation, role play or facilitated practice to accompany the online material. They valued the resource as a knowledge-building tool, but felt that speaking skills would be further developed through rehearsal.

*Something like a simulated conversation at the end would make it feel more complete*,* because then you’d be trying out what you had learned [P2].*

Another said,

*The website gave you the knowledge*,* but I think practising it out loud would be the next step for building confidence [P11].*

Overall, this theme shows that students described movement from understanding ACP in theory towards recognising, discussing and contributing to it in placement, while still identifying a need for further practical rehearsal.

### Theme 3: spreading the learning

A notable feature of the interviews was that the resource often did not remain with the student alone. Instead, many participants described it being shared with others in the nursing home and beyond. In several placements, staff became aware that students were completing ACP-related learning during work time and asked about the resource. Students described explaining what it was, why they were doing it, and how it related to care home practice. Because the resource was seen as directly relevant to the setting, staff interest was often immediate.

*They were asking what I was doing*,* and when I explained it was about ACP in care homes*,* they said they’d like to look at it too because it sounded useful for them as well [P4].*

Another reflected,

*Once they realised it was practical for their setting and not just some university thing*,* they were really interested in it [P12].*

These accounts show that students often became the first point through which staff encountered the resource.

In some placements, this interest moved beyond simple curiosity. Students described actively showing the website to staff, discussing it with nurses or care assistants, and passing the link on to others. A small number gave examples of managers encouraging wider uptake within the home. In one interview, a participant recalled that a manager viewed the resource as something staff should be using and wanted others to have access to it. In another, a flyer or printed information about the site was displayed at the nurses’ station.

*The manager said it was exactly the kind of thing staff should be doing and wanted everyone to have the link [P8].*

Another said,

*They actually printed it out and put it up so people could go on and do it if they wanted [P15].*

These examples suggest that, in some settings, students’ access to the resource prompted a wider spread of interest within the placement environment.

Participants did not describe themselves as formally teaching staff. Rather, they presented their role as introducing or signposting something useful. Several students were careful to distinguish between sharing the resource and instructing others.

*I didn’t feel like I was teaching anybody or telling them what to do*,* but I did feel like I was bringing something useful into the home that maybe people hadn’t seen before [P1].*

This shows that students understood their own position as learners while still recognising that they could pass on relevant material. Their role was often modest, but they nonetheless described helping the resource travel from university-linked learning into the care home environment. In a smaller number of interviews, students described pointing people towards the family-facing parts of the website. These accounts were less detailed, but they suggested that some participants recognised the resource as having potential relevance beyond staff learning. Where this happened, students appeared to view the family material as another practical part of the site that could support conversations around future care. The resource was therefore not always seen as something for students alone, but as something with wider usefulness within the nursing home context.

Sharing also extended beyond the placement site itself. Some students described circulating the link among peers, particularly other students on placement. This happened through informal conversations and through student group chats.

*I put it into our WhatsApp group because I thought other people on placement would probably find it helpful too*,* even if they weren’t in a nursing home” [P6]. Another participant described posting the link on social media because they felt it was a worthwhile educational resource [P13].*

These examples show that students sometimes continued sharing the resource through their own networks after seeing value in it themselves. Importantly, students’ accounts did not suggest that this sharing happened in every placement or that it always led to sustained use by others. Nor did participants claim that staff widely completed the resource or that organisational practice changed as a direct result. The significance of the theme lay instead in the repeated description of the resource being passed on informally to other people. Across interviews, students described the resource moving outwards from their own learning into wider conversations and local interest. This pattern was unplanned, but recurred often enough to stand out clearly in the analysis.

Overall, this theme shows that the resource was frequently shared beyond the individual student. Students described introducing it to staff, managers, peers and, in some cases, family members. In doing so, they acted as a route through which the resource reached others in practice settings. The accounts do not demonstrate formal implementation, but they do show that participants often perceived the resource as useful enough to pass on, and that this sharing formed a meaningful part of their experience of using it during placement.

## Discussion

ACP is a core component of high-quality, person-centred end-of-life care, particularly in nursing homes where residents commonly live with multimorbidity, frailty and cognitive impairment [10, 51]. International consensus describes ACP as an ongoing process through which individuals articulate values, goals and preferences for future care, which are then discussed, reviewed and communicated over time [2, 52]. This is especially important where progressive frailty, dementia and fluctuating decisional capacity require families and staff to navigate uncertainty about deterioration and future care [6, 8]. Yet healthcare professionals continue to report variable confidence and preparedness in initiating and sustaining ACP conversations [10, 52–54].

This study suggests that a care-home-specific digital e-resource may support undergraduate nursing students to recognise, understand and engage with ACP-related issues during placement. The findings do not demonstrate competence or independent proficiency; rather, they indicate greater perceived readiness, clearer recognition of ACP in everyday care and increased willingness to participate appropriately in relevant discussions. These findings are consistent with Social Cognitive Theory, particularly self-efficacy, which sensitised the study [35, 36]. Students described the resource as providing structure, language and conceptual clarity for a topic that can feel unfamiliar and daunting early in training. It may therefore have reduced uncertainty and avoidance while supporting cognitive preparation and perceived capability. However, students did not suggest that it resolved the complexity of real conversations or that online learning alone could produce comprehensive confidence. Given their limited prior ACP education, the resource appears to have complemented existing teaching through contextualised preparation for practice.

This is relevant to palliative care education. In nursing homes, ACP is embedded within recognition of deterioration, communication about goals of care, family support and planning during progressive illness and dying [51, 55, 56]. Preregistration students may encounter these issues early, yet educational preparation remains variable in content, timing and pedagogical approach [17, 57]. Whereas much of the literature has focused on simulation, classroom teaching or curriculum review, this study examined a focused resource used during placement in the setting to which its content applied. Context-specific digital learning may therefore help students connect palliative care principles with nursing home practice.

The self-care content was also valued. Palliative care education often prioritises communication, ethical reasoning and clinical decision-making, with less attention to the emotional labour associated with deterioration, dying and family distress. Participants’ accounts suggest that acknowledging emotional impact was important, particularly when exposure to decline and death was new. Although the resource cannot be said to have fully addressed this dimension, ACP education may be strengthened by attending to the emotional as well as procedural and communicative demands of care.

The findings also show that the acceptability of digital education depends on both content and implementation conditions. Protected time appeared to legitimise learning and may have supported engagement, whereas asynchronous delivery did not suit all students. Participants identified variable motivation, a preference among some learners for more direction, and a need for greater interactivity. This aligns with evidence that e-learning can support knowledge and confidence, but that effectiveness is influenced by pedagogical design, learner support and opportunities for application [26–28, 58]. The e-resource therefore provided a useful foundation rather than a complete educational response.

Students’ calls for simulation, role play and facilitated discussion highlighted the distinction between understanding ACP conceptually and communicating in emotionally complex situations. ACP requires knowledge, communicative judgement, relational sensitivity and the ability to respond in real time [59, 60]. Digital ACP education may therefore be most useful within a multimodal approach combining online content with rehearsal, reflection and guided discussion. This is consistent with literature emphasising interactive and practice-oriented approaches for translating theoretical learning into applied communication [18, 20, 21, 23, 27, 28, 31, 61–64]. The findings do not establish the superiority of one model, but indicate that students viewed online learning as preparatory rather than sufficient.

Comparison with the original *Necessary Discussions* evaluation is informative. Previous work with nursing home staff found the resource acceptable and associated with improved knowledge and confidence, although time pressures and implementation barriers remained [9, 10]. The present study suggests that it may also have value in undergraduate education when aligned with students’ stage of learning and placement context. Students used the material to interpret ongoing care, participate more appropriately in discussions and, in some cases, pursue further information. Its usefulness was closely linked to its relevance to nursing home practice and use during placement.

Finally, students described sharing the resource with staff, managers, peers and occasionally family members. These accounts do not demonstrate formal implementation, sustained organisational change or improved ACP practice, but suggest a possible route for extending the reach of educational interventions. This is relevant to knowledge translation literature highlighting the difficulty of extending palliative approaches beyond specialist services into general care settings [65]. Through the lens of boundary crossing, students may have acted as informal boundary crossers between university and nursing home communities of practice by introducing and sharing an evidence-informed resource across settings [65–68]. Although informal, variable and modest in scale, this points to a potentially under-recognised route to impact through preregistration education.

### Strengths and limitations

This study used a qualitative descriptive design to generate practice-relevant findings grounded in students’ experiences of a care-home-specific e-resource during placement. An interpretivist orientation and Social Cognitive Theory, particularly self-efficacy, provided conceptual coherence across the study aim, interview guide and interpretation. Reporting followed COREQ, while reflexive thematic analysis involved iterative coding and theme development by GM and SC, supported by reflexive discussion and an audit trail. A further strength was examining the resource within the nursing home context to which it directly applied. Individual interviews with 16 first-year nursing students also generated sufficient experiential depth to address the study aim.

Several limitations should be considered. The study was conducted within one educational institution, which may limit transferability. Voluntary participation introduced possible self-selection bias, as students with greater interest in palliative care, care home practice or digital learning may have been more likely to participate. Non-participants may therefore have held different views. Although the age and gender profile of interviewees broadly reflected the wider group who volunteered to be contacted, further comparison was not possible because additional background data were unavailable.

Findings were based on self-reported perceptions rather than direct observation or objective measures of behaviour. Demographic data beyond age group, gender and nursing field were not collected, nor were objective engagement data such as time spent or completion rates. Small numbers across adult, learning disability and mental health nursing also prevented meaningful comparison between fields. Future research should examine whether experiences differ by nursing field.

GM was not involved in placement assessment but was known to students academically and had introduced the resource within nurse education, which may have influenced interview dynamics or willingness to express criticism. Finally, the absence of longitudinal follow-up means it is unclear whether reported preparedness was sustained or translated into later practice.

## Conclusion

This study suggests that a digital, care-home-specific e-resource may support first-year nursing students during nursing home placements. Participants described the resource as accessible, relevant and appropriate to their stage of learning, helping to make ACP more visible and understandable in practice. Across the themes of *Building Confidence*, *Putting Learning into Practice* and *Spreading the Learning*, students reported greater perceived readiness to engage with ACP, examples of applying learning during placement and informal sharing of the resource with others.

Although the study does not demonstrate behavioural competence or sustained practice change, it supports the cautious integration of theoretically informed digital ACP education within preregistration curricula, particularly when aligned with placement context and supplemented by rehearsal, reflection and discussion.

## Data Availability

The datasets used and/or analysed during the current study are available from the corresponding author on reasonable request.
